# In Vitro Spectrophotometry of Tooth Discoloration Induced by Tooth-Colored Mineral Trioxide Aggregate and Calcium-Enriched Mixture Cement

**DOI:** 10.7508/iej.2015.04.003

**Published:** 2015

**Authors:** Marjan Arman, Zohreh Khalilak, Moones Rajabi, Ehsan Esnaashari, Keyvan Saati

**Affiliations:** a *Department of Endodontics, Dental Branch, Islamic Azad University of Medical Sciences, Tehran, Iran; *; b * Department of Oral and Maxillofacial Pathology, Dental school, Kerman University of Medical Sciences, Kerman, Iran; *; c * Department of Restorative Dentistry, Dental Branch, Islamic Azad University of Medical Sciences, Tehran, Iran*

**Keywords:** Calcium-Enriched Mixture, CEM Cement, Crown Discoloration, Mineral Trioxide Aggregate, MTA, Spectrophotometer

## Abstract

**Introduction:**

There are numerous factors that can lead to tooth discoloration after endodontic treatment, such as penetration of endodontic materials into the dentinal tubules during root canal treatment. The aim of this *in vitro* study was to compare discoloration induced by tooth colored mineral trioxide aggregate (MTA) and calcium-enriched mixture (CEM) cement in extracted human teeth.

**Methods and Materials:**

Thirty two dentin-enamel cuboid blocks (7×7×2 mm) were prepared from extracted maxillary central incisors. Standardized cavities were prepared in the middle of each cube, leaving 1 mm of enamel and dentin on the labial surface. The specimens were randomly divided into two study groups (*n*=12) and two positive and negative control groups (*n*=4). In either study groups the cavities were filled with MTA or CEM cement. The positive and negative control groups were filled with blood or left empty, respectively. The cavities were sealed with composite resin and stored in normal saline. Color measurement was carried out by spectrophotometry at different time intervals including before (T_0_), and 1 week (T_1_), 1 month (T_2_) and 6 months (T_3_) after placement of materials. Repeated-measures ANOVA was used to compare the discoloration between the groups; the material type was considered as the inter-subject factor. The level of significance was set at 0.05.

**Results:**

No significant differences were detected between the groups in all time intervals (*P*>0.05).

**Conclusion:**

Tooth discoloration was similarly detectable with both of the two experimental materials.

## Introduction

Tooth discoloration is a common problem with endodontic materials [1], resulting in dissatisfaction in 31.6%-57% of the patients [2]. The tooth staining potential of several endodontic materials has been demonstrated [3-6]. Some studies insist that tooth discoloration is due to endodontic sealers [7, 8]. Studies focusing on discoloration have also focused on biomaterials like calcium-enriched mixture (CEM) cement and mineral trioxide aggregate (MTA) that are sometimes used in tooth crowns during various vital pulp therapies including pulp capping [9-11]. 

CEM cement is a bio-regenerative material consisting of different calcium compounds (*i.e.* calcium oxide, calcium phosphate, calcium carbonate, calcium silicate, calcium sulfate, calcium hydroxide, and calcium chloride) [12]. Clinical applications of CEM cement are similar to MTA [12]. CEM has good handling properties and is able to produce hydroxyapatite with its endogenous and exogenous ion sources [13]. A study on dogs showed that after pulp capping with MTA and CEM, pulp histological response was similar [13].

The principal constituents of the gray-colored formulation of MTA are tricalcium silicate, bismuth oxide, dicalcium silicate, tricalcium aluminate, tetra calcium aluminoferrite and calcium sulfate dehydrate [3]. Tooth-colored formulation of MTA (TC-MTA) lacks tetra calcium aluminoferrite [3]. According to recent studies, there are no significant differences between gray and TC-MTA in terms of tooth discoloration [9, 10]. 

**Figure 1:**
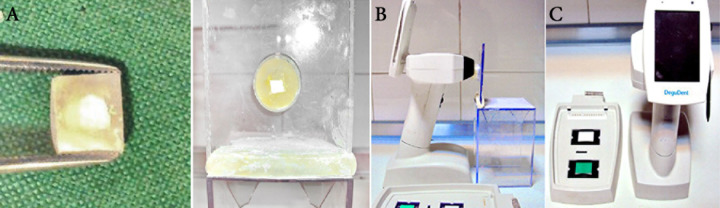
*A) *Specimen preparation; a cylindrical-shaped hole measuring 2.5×2.5×1 mm was drilled with a bur in the middle of each specimen up to 1 mm deep in the labial tooth structure, *B)* Fixed chamber made of putty and *C) *Spectrophotometer

Since the discoloring effects of new endodontic materials have only been rarely mentioned in literature [1] and there is no published data available on discoloration effects of CEM cement, the present *in vitro* study was conducted to analyze and compare the discoloration effects of TC-MTA and CEM cement on human extracted teeth.

## Materials and Methods

***Specimen preparation***

Thirty-two intact caries-free human maxillary central incisors without any cracks and previous root canal therapy were used. The teeth were cleaned with a scaler to dislodge debris and calculus followed by a rubber cup and pumice to remove remaining debris. All specimens went under color determination with spectrophotometer and the three values were specified (T_0_). Then a cuboid enamel-dentin block (with 7×7×2 mm diameters) was prepared from the middle of each crown using a diamond-coated disc (Intensiv SA, Grancia, Switzerland). The dimension of each block was standardized with a caliper (Iwanson,Ustomed, Tuttlingen, Germany). Then, a cylindrical-shaped hole measuring 2.5×2.5×1 mm was drilled with a bur (Dentsply Maillefer, Ballaigues, Switzerland) in the middle of each specimen so that 1 mm of the labial tooth structure was left on each cube (Figure 1A). The specimens were immersed in 5.25% sodium hypochlorite (NaOCl) and then in 17% EDTA for 1 min to remove the smear layer. Finally the specimens were rinsed with normal saline.

The cubes were randomly assigned into two experimental groups (*n=*12) in which the cavities were filled with either TC-MTA (Angelus MTA, Angelus, Londrina, Paraná, Brazil) or CEM cement (BioniqueDent, Tehran, Iran). In the positive and negative control groups (*n*=4) the cavities were either filled with blood or left empty, respectively. 

The materials were prepared according to the manufacturers’ recommendations and placed into the cavity with an applicator. In the positive control group, 0.5-10 µl of fresh human blood obtained from a volunteer was placed on top of the material with a pipette (Eppendorf AG, Hamburg, Germany). The cavities were temporarily covered by wet cotton pellet and Coltozol (Coltene, Altstatten, Switzerland). After 24 h of keeping the teeth in incubator with 37^°^C temperature, the cavities were sealed with a light-cured self-adhesive resin material (A1 shade Flowable, Prim-Dent, USA). Subsequently, every specimen was placed into a single tube containing normal saline. To simulate the conditions of the oral cavity, the tubes were stored at 37^°^C in an incubator. 

***Spectrophotometry***

Color measurements were carried out in a dark room with a spectrophotometer (Dentsply International Shade Pilot, Degudent, Germany) under standardized conditions. Then the teeth were removed from their individual tubes and placed on a fixed chamber made of putty, (Speedex, Colten, Switzerland) to conduct post-operative spectrophotometry at different time intervals including: T_1_ (one week), T_2 _(1 month) and T_3_ (6 months) (Figure 1B and C).

Before measuring color changes, the instrument was calibrated on green and white surfaces. The degree of discoloration for each sample was measured three times and the mean value was considered the final measurement. According to Cal *et al.* [14], Δ*E* is the amount of color change if *L* is the lightness and *A* (red as *A*^+^ and green as *A*^-^) and *B *(yellow as *B*^+^ and blue as *B*^-^) determine the chroma. 

The color difference was evaluated according to the following formula: Δ*E*=[(Δ*L*)²+(Δ*A*)²+(Δ*B*)²]/2. 

Data was analyzed using the SPSS software (SPSS version 22, SPSS, Chicago, IL, USA). Repeated-measures ANOVA and HSD Tukey’s tests were used to compare the color indexes between the four groups at different time intervals by considering the type of the material as the inter-subject factor. Statistical significance was defined at 0.05.

## Results

An intra-group comparison of color measurements of TC-MTA over time revealed that changes of Δ*Ε* at different time intervals (T_0_ to T_3_) was significant (*P*=0) (multivariate tests). Also similar comparison of color measurements of CEM cement shows that changes were significant (*P*=0) (Friedman test). The lightness of both materials significantly decreased with time (*P*= 0). With regards to chroma, *A* increased significantly with time in both materials (*P*=0 and *P*=0.03 for TC-MTA and CEM cement, respectively). Meanwhile, *B* decreased significantly with time (*P*=0.015 and *P*=0 for TC-MTA and CEM cement, respectively). Table 1 shows the changes of color determinants in different times after using the specific materials. Two of the samples from positive control and CEM cement experimental groups, were excluded due to fracture. 

***After 1 week (T***_1_ ***)***

There were no significant differences in discoloration among the four groups. The Δ*Ε* value for TC-MTA and CEM cement was 0.6465 and 0.2750, respectively. For the positive control this amount was 0.3204 and for negative control it reached 0.3768. 

***After 1 month (T***_2_ ***)***

Significant differences were detected between CEM cement and positive control groups (*P*=0.034). Moreover, there was no significant difference between CEM cement and TC-MTA groups.

*ΔA*: There was no significant difference between TC-MTA and CEM cement (*P*=0.50). TC-MTA showed significantly greater red staining compared to blood (*P*=0.04) and the negative control group (*P*=0.03)

*ΔB*: No significant differences were detected between TC-MTA and CEM cement (*P*=0.92)

*ΔL*: MTA and CEM cement revealed no significant differences (*P*=0.97).

***After 6 months*** (T_3_)

The negative control group showed the least amount of discoloration, validating the study. There was no significant difference in discoloration between CEM cement and TC-MTA (*P*=0.89). However, neither CEM cement, nor TC-MTA revealed a significant difference compared with the positive control group (*P*=0.51); the positive and negative control groups exhibited significant differences (*P*=0.002). 

The severity of tooth discoloration was similar after 6 months with TC-MTA, CEM cement and blood. 

*ΔA:* There was no significant difference between TC-MTA and CEM cement (*P*=0.50). TC-MTA showed significantly greater red staining (*A*^+^) compared to blood (*P*=0.04) and the negative control group (*P*=0.05). However, there was no such difference between CEM cement and TC-MTA, and CEM cement and the positive control groups.

*ΔB*: No significant differences were detected between TC-MTA and CEM cement groups and the control groups. However, the difference between the control groups was significant (*P*=0.034). In conclusion, blood resulted in less yellow discoloration (*B*^+^).

*ΔL:* MTA, CEM cement and blood revealed no significant differences, although, an obvious difference was found between these materials and the negative control (*P*=0.04). The current study showed that MTA, CEM cement and blood resulted in a decrease in *L *variable (degree of lightness).

## Discussion

This *in vitro* study compared the discoloration potential of TC-MTA and CEM cement in human teeth. No study has focused on the discoloration potential of CEM cement, however there are many studies that have investigated MTA. Incisor teeth were selected because of their size that enables preparation of dentine cubes with desired dimensions. A cuboid enamel-dentin block was prepared using each crown with a diamond-coated disc. The dimensions of blocks were standardized. A cylindrical-shaped whole measuring 2.5×2.5×1 mm was drilled with a bur in the middle of each specimen to leave 1 mm of the labial tooth structure. This structure was made similar to the study by Lenherr *et al.* [1].

Recently it has been suggested that the smear layer might be an obstacle for irrigation [15]. In addition, it has been reported that the smear layer can significantly decrease dentin permeability [1]. Therefore, in this study the samples were soaked in 17% EDTA and 5.25% NaOCl each for 1 min to remove the smear layer [16].

Color change can be calculated by CIE LAB color system which is approved by American Dental Association (ADA) and is the most popular and accurate system used at present [17]. According to Dozić *et al. *[18], spectrophotometer is the most reliable instrument both *in vitro* and *in vivo*.

**Table 1:** Changes of color determinants in different times after using the specific materials

	**T**_1 _ **(one week)**				**T**_2 _ **(one month)**				**T**_3 _ **(six months)**			
	**ΔΕ**	**ΔL**	**ΔA**	**ΔB**	**ΔΕ**	**ΔL**	**ΔA**	**ΔB**	**ΔΕ**	**ΔL**	**ΔA**	**ΔB**
**TC-MTA**	0.6465	0.1417	0.1167	0.0333	6.5429	3.9000	0.0083	0.6833	11.4810	8.0583	2.1333	5.2250
**CEM**	0.2750	0.0083	0.0083	0.0417	3.8503	2.3364	0.4636	0.3091	10.1299	7.2636	1.3727	6.4091
**Positive control**	0.3204	0.0250	0.1000	0.2500	11.7026	8.1250	0.1750	6.1250	15.1635	10.4250	0.0750	10.4500
**Negative control**	0.3768	0.0667	0.1000	0.0667	0.2718	0.2333	0.6333	0.1800	0.1270	0.1000	0.3333	0.3667

Measurements that are carried out by a spectrophotometer under standardized light conditions are probably more reliable and accurate than visual color discrimination. Despite the possible uncertainty in shade measurement with the use of a spectrophotometer [17], we decided to utilize spectrophotometer due to its availability and intra-examiner reliability.

In the research by Parson *et al. *[7], discoloration was most evident at 9- and 12-month intervals, whereas the 1- and 3-month intervals exhibited minimal or no discoloration, similar to the present study. 

Lenherr *et al.* [1] found that after 12 months, progressive discoloration was observed with TC-MTA combined with blood. In addition, gray MTA showed severe discoloration immediately after placement. After a slight increase during the first week, the value* ΔE* remained stable at this high level. Felman and Parashos [19] revealed that all the teeth showed some form of discoloration when restored with TC-MTA. The presence of blood within the canal exacerbated discoloration. In their study, all the coronal tooth parts were filled with TC-MTA, which might have led to exaggerated discoloration during the study procedure. 

From the results of the present study, it can be concluded that there was no significant difference between the discoloration induced by TC-MTA and CEM cement. Both materials resulted in discoloration in 6 months (*ΔE*>3.3) [17]. Therefore, according to the results of the present study, it took 6 months for the materials to penetrate into the dentinal tubules. 

Similar to our study, Jang *et al. *[20] showed that discoloration increases with time after using MTA. However they did not consider a positive control and their results cannot be compared with ours [20].

According to Camilleri [21], immersion of TC-MTA and bismuth oxide in sodium hypochlorite resulted in a dark brown discoloration. Considering that CEM cement does not contain bismuth oxide, it is logical to use CEM cement in cases where using sodium hypochlorite is inevitable.

In the research by Felman and Parashos [19] the 35-day results is similar to T_2_ results in our study. After one month, the difference between TC-MTA and positive control group was significant in both studies.

The gray color of the original MTA caused severe tooth discoloration. In order to resolve this issue, TC-MTA was introduced [6]. Asgary *et al.* [3] reported that the major differences in the chemical compositions of TC-MTA and gray MTA were related to the concentrations of Al_2_O_3_, MgO and FeO. Set TC-MTA contains only 9% of the iron oxide content of gray MTA [3, 22]. The difference between tooth discolorations caused by the two materials is related to the difference in their chemical compositions. The new formulation was thought to be more suitable for anterior esthetic region of the oral cavity, [23] although slight discoloration may still result [21]. 

Comparison between the chemical compositions of the two kinds of MTA with CEM, shows that in contrast to MTA, CEM has no Fe and bismuth oxide in its content [24]. The present study revealed that CEM cement and TC-MTA changed the tooth color similarly. In other words, although CEM cement does not contain Fe and bismuth oxide, it may still cause discoloration similar to MTA. Tooth discoloration after using CEM cement might be related to its chemical compounds, such as calcium oxide, calcium phosphate, calcium carbonate, calcium silicate, calcium sulfate, calcium hydroxide and calcium chloride. However, the exact etiology and mechanism of discoloration is yet to be discovered and assessed.

## Conclusion

There was a similar level of clinically observable tooth discoloration detected using either tooth-colored MTA or CEM cement. Application of both biomaterials should be considered with high level of caution, especially in the esthetic zones.
